# Computing all persistent subspaces of a reaction-diffusion system

**DOI:** 10.1038/s41598-023-44244-x

**Published:** 2023-10-11

**Authors:** Stephan Peter, Linus Woitke, Peter Dittrich, Bashar Ibrahim

**Affiliations:** 1 Department of Basic Sciences, Ernst-Abbe University of Applied Sciences Jena, Carl-Zeiss-Promenade 2, 07745 Jena, Germany; 2 Department of Mathematics and Computer Science, Friedrich Schiller University Jena, Fürstengraben, 07743 Jena, Germany; 3 Department of Mathematics & Natural Sciences and Centre for Applied Mathematics & Bioinformatics, Gulf University for Science and Technology, 32093 Hawally, Kuwait; 4 European Virus Bioinformatics Center, Leutragraben 1, 07743 Jena, Germany

**Keywords:** Computational science, Applied mathematics

## Abstract

An algorithm is presented for computing a reaction-diffusion partial differential equation (PDE) system for all possible subspaces that can hold a persistent solution of the equation. For this, all possible sub-networks of the underlying reaction network that are distributed organizations (DOs) are identified. Recently it has been shown that a persistent subspace must be a DO. The algorithm computes the hierarchy of DOs starting from the largest by a linear programming approach using integer cuts. The underlying constraints use elementary reaction closures as minimal building blocks to guarantee local closedness and global self-maintenance, required for a DO. Additionally, the algorithm delivers for each subspace an affiliated set of organizational reactions and minimal compartmentalization that is necessary for this subspace to persist. It is proved that all sets of organizational reactions of a reaction network, as already DOs, form a lattice. This lattice contains all potentially persistent sets of reactions of all constrained solutions of reaction-diffusion PDEs. This provides a hierarchical structure of all persistent subspaces with regard to the species and also to the reactions of the reaction-diffusion PDE system. Here, the algorithm is described and the corresponding Python source code is provided. Furthermore, an analysis of its run time is performed and all models from the BioModels database as well as further examples are examined. Apart from the practical implications of the algorithm the results also give insights into the complexity of solving reaction-diffusion PDEs.

## Introduction

Understanding a system and predicting its behavior is in general a complex problem because a system’s behavior as a whole results in general from many non-linear interactions of its components. A particular challenge is to infer a system’s behavior from its structure. In this work the structure of a system is described as a reaction network, that is a set of reaction rules over a set of molecular species^1^. Reaction network models are not only used in chemistry but also in various other disciplines such as biology and genetics^2–6^, physics^7^, virology^8–11^, computer science^12^, ecology^13^, economy^14^, or even social sciences^15^.

Chemical organization theory offers a way to relate the structure of a reaction network to the potential dynamics of a related dynamical system^16,17^. In particular, every fixed point of a system of ordinary differential equations is an organization^16,18^. The basic theory does not consider a spatial or temporal distribution of the system’s components. Nevertheless, the eukaryotic cell, for example, accomplishes its dynamics through a spatial arrangement of its compartments. Different parts of the cell perform different reactions to create an overall cycle attaining self-maintenance. The need for such separations can be seen easily upon the effect of the peroxisomes, which isolate strong oxidants to avoid harming any functioning organic material. Spatial separation also operates at many other scales, such as in the development of different organs in multi-cellular organisms. On the other hand, temporal separation can be seen in many models exhibiting periodical behavior. Synchronization of several transitive states can lead to a stable solution^19^.

To incorporate such spatial and temporal separation in chemical organization theory, Peter et al.^20^ recently introduced the concept of a distributed organization (DO). A distributed organization is a subset of species that achieves overall self-maintenance by separation into suitable compartments^20,21^. As a generalization of the above-mentioned relation of fixed points and organizations, it was also shown that every persistent subspace of a PDE system is a DO^20,21^. This represents a method for analyzing dynamical systems that are comparable to commonly used fixed point analyses^22^. While a mathematical theory has been introduced, an algorithm is not available so far. A particular problem is that the separation into suitable compartments is not trivial and has to be found by the algorithm.

This paper is structured as follows. In the “Preliminaries” Section, an example reaction network is used to introduce Chemical organization theory and DOs. The “Results” Section consists of three parts. First, the main focus is shifted from species to reactions by considering subsets of organizational reactions belonging to a DO. Using this and the persistence theorem (Theorem 3.25 in^20^) a theorem is derived that states that for all bounded solutions of a reaction-diffusion system the set of potentially persistent reactions of an a reaction-diffusion system always forms a set of organizational reactions of the underlying reaction network. Then the fundamental new concepts of an elementary reaction closure and maximal compartment are described mathematically and applied to the same example reaction network. The elementary reaction closures reflect the dependence of reactions on one another, whereas maximal compartments describe the minimal number of compartments required for a subspace to be persistent. After that, all functions building up the algorithm are described in detail and their run time is studied. The core of the algorithm uses mixed-integer linear programming solver, which can be exchanged. Finally, we apply the algorithm to several example models including all models of the BioModels database.

An implementation of the algorithm developed in this word is available in the GitHub https://github.com/WoitkeL/dorganalysis.

## Preliminaries

In this section, the definitions and main results from^20^ are introduced, which set the stage for the algorithm presented in this work. A reaction network $({\mathscr {S}},{\mathscr {R}})$ consists of a finite set ${\mathscr {S}}$ of $n\in {\mathbb {N}}$ species as well as a finite set ${\mathscr {R}}$ of $m\in {\mathbb {N}}$ reactions describing the interactions between the species. Note that both, ${\mathscr {S}}$ and ${\mathscr {R}}$, are assumed to be finite sets throughout this work.

### Introductory example

As an example, we consider the following reaction network describing the role of microRNAs in osteoarthritis^23^. It is a micro-RNA transcription-factor interaction model. The set of species is

$$\begin{aligned} TF1&: \text { Transcription factor 1}\\ TF2&: \text { Transcription factor for miR synthesis}\\ miR&: \text { micro RNA}\\ miR\_gene&: \text { gene of micro RNA}\\ Sink&: \text { EmptySet}\\ Signal&: \text { signal of TF1 transcription}\\ miR\_gene\_TF2&: \text { mir\_gene\_TF2 complex}\\ miR\_gene\_TF1&: \text { mir\_gene\_TF1 complex}\\ TF1\_mRNA&: \text { TF1\_mRNA complex} \end{aligned}$$

and the set of reactions

$$\begin{aligned} r_1&: miR\_gene + TF1 \longrightarrow miR\_gene\_TF1&\text {(miR-gene TF1 binding)}\\ r_2&: miR\_gene\_TF1 \longrightarrow miR\_gene + TF1&\text {(miR-gene TF1 release)}\\ r_3&: miR\_gene + TF2 \longrightarrow miR\_gene\_TF2&\text {(miR-gene TF2 binding)}\\ r_4&: miR\_gene\_TF2 \longrightarrow miR\_gene + TF2&\text {(miR-gene TF2 release)}\\ r_5&: miR\_gene\_TF2 \longrightarrow miR\_gene\_TF2 + miR&\text {(miR-synthesis)}\\ r_6&: miR \longrightarrow Sink&\text {(miR-degradation)}\\ r_7&: Signal \longrightarrow Signal + TF1\_mRNA&\text {(TF1-transcription)}\\ r_8&: TF1\_mRNA \longrightarrow Sink&\text {(TF1-mRNA-degradation)}\\ r_9&: TF1\_mRNA + miR \longrightarrow miR&\text {(TF1-mRNA-deg via miR)}\\ r_{10}&: TF1\_mRNA \longrightarrow TF1\_mRNA + TF1&\text {(TF1-translation)}\\ r_{11}&: TF1 \longrightarrow Sink&\text {(TF1-degradation)}, \end{aligned}$$

thus $n=9$ and $m=11$ for this example.

Generally, the equation of reaction number *j* can be described by

1 $$\begin{aligned} \sum _{i=1}^n a_{ij} s_i \rightarrow \sum _{i=1}^{n} b_{ij} s_i \end{aligned}$$

with natural numbers $a_{ij},b_{ij},\;j=1,\ldots ,m$ which can be zero. For the reaction $r_j$ the set of species $s_i$ with $a_{ij}>0$ is called support of $r_j$, shortly $supp(r_j)$, and the set of species $s_i$ with $b_{ij}>0$ the products of $r_j$, shortly $prod(r_j)$. For a subset $S\subseteqq {\mathscr {S}}$ of species, we say that it *supports* a reaction $r\in {\mathscr {R}}$, if $supp(r)\subseteqq S$.

A reversible reaction is a reaction in which the conversion of reactants to products and the conversion of products to reactants occur simultaneously. In this work, reversible reactions are represented by a pair of two separate reactions, which are both active or inactive at the same time. From the set of reactions the so-called stoichiometric matrix $N\in {\mathbb {R}}^{n\times m}$ with its elements $n_{ij}=b_{ij}-a_{ij},\,i=1,\ldots ,n,\, j=1,\ldots ,m$ is derived.

### Chemical organization theory (COT)

#### Definition 1

(Closure of a subset of species) Given a reaction network $({\mathscr {S}},{\mathscr {R}})$ and a subset $S\subseteq {\mathscr {S}}$ of species. We define the set operation

2 $$\begin{aligned} clos_1(S)\equiv S\cup \{s_i\in {\mathscr {S}}:\,\exists r_j\in {\mathscr {R}}:\, supp(r_j)\subseteq S,\, b_{ij}>0\}, \end{aligned}$$

that is, the set of species from *S* together with all species, that are produced by the reactions, which are active on *S*. From this, we define a monotonously increasing sequence of sets

$$\begin{aligned} clos_1^0(S)= & {} S,\\ clos_1^1(S)= & {} clos_1(S),\\ clos_1^2(S)= & {} clos_1(clos_1(S)),\\ clos_1^3(S)= & {} clos_1(clos_1(clos_1(S))),\\&\ldots&\\ clos_1^{k_{min}+1}(S)= & {} clos_1(clos_1^{k_{min}}(S)), \end{aligned}$$

where $k_{min}=\min \{k\in {\mathbb {N}}_0:\,clos_1^{k+1}(S)=clos_1^k(S)\}.$ Since the set of species and the set of reactions is finite, $k_{min}$ is finite, and thus also the set

3 $$\begin{aligned} clos(S)\equiv clos_1^{k_{min}}(S), \end{aligned}$$

which we call the closure of *S*.

Generally, flux vectors ${\textbf{v}}\in {\mathbb {R}}_+^m$, where

$$\begin{aligned} {\mathbb {R}}_+^m\equiv \{v\in {\mathbb {R}}^m:\; v_i\ge 0,\,i=1,\ldots ,m\}, \end{aligned}$$

are used in dynamical systems to describe the intensity of each reaction for a given state of the system. Depending on the present species in that state, not all flux vectors are feasible, because it is assumed that a reaction is active if and only if all the species of its support are present.

#### Definition 2

(Feasible flux and inflow reaction) For a given subset $S\subseteqq {\mathscr {S}}$ of species, a vector $v\in {\mathbb {R}}_+^m$ $v_r$ is called *feasible flux* (with respect to *S*) if and only if for all reactions $r\in {\mathscr {R}}$.

4 $$\begin{aligned} v_r > 0 \Leftrightarrow supp(r)\subseteqq S. \end{aligned}$$

Furthermore, a reaction $r \in {\mathscr {R}}$ that has empty support is called *inflow reaction* as it is always active, that is, $v_r>0$ for every feasible flux *v*.

#### Definition 3

(Closedness, self-maintenance and organizations) Given a reaction network $({\mathscr {S}},{\mathscr {R}})$ and a subset $S\subseteq {\mathscr {S}}$ of species then we call *S*.

1. *self-maintaining* if there is a feasible flux *v* with respect to *S* such that
  5 $$\begin{aligned} N v\ge 0, \end{aligned}$$
  that is, all elements of *Nv* are equal or greater than zero,
2. *closed* if
  6 $$\begin{aligned} clos(S)=S, \end{aligned}$$
3. *organization* if it is self-maintaining and closed.

### Distributed organizations (DOs)

The previously defined organizations were generalized towards so-called distributed organizations (DOs), which are introduced and broadly discussed in^20,21^.

#### Definition 4

(Distributed organizations (DOs)) Given a reaction network $({\mathscr {S}},{\mathscr {R}})$, a subset $D\subseteq {\mathscr {S}}$ is a DO (through a vector ${\hat{v}}\in {\mathbb {R}}_+^m$) if and only if there are *k*, $k\in {\mathbb {N}}$, pairwise different subsets (which we call “compartments” according to nomenclature of systems biology) $S_1,\ldots ,S_k\subseteq D$ with

7 $$\begin{aligned} D=\cup _{i=1}^k S_i \end{aligned}$$

such that

1. each $S_i,\;i=1,\ldots ,k,$ is *closed*;
2. there is a vector ${\hat{v}}\in {\mathbb {R}}_+^{m},\, {\hat{v}} \ge 0,$ such that
  8 $$\begin{aligned} N{\hat{v}}\ge 0; \end{aligned}$$
3. and there is a *feasible flux* ${\hat{v}}^i\in {\mathbb {R}}_+^{m},\, {\hat{v}} ^i\ge 0,$ with respect to each subset $S_i,\;i=1,\ldots ,k$, with
  9 $$\begin{aligned} {\hat{v}}=\sum _{i=1}^k {\hat{v}} ^i . \end{aligned}$$

Collectively, we call the Eqs. (8) and (9) the self-maintenance property of a DO. We say “*D* *is distributed to the compartments* $S_i$”, “the compartments $S_i$ form a compartmentalization (or distribution) of *D*” or “$S_i$ *is a compartment of* *D*”. When listing the elements of the subsets $S_i,\,i=1,\ldots ,k,$ of species, we use a vertical notation, for example, if *D* is distributed to $S_1 =\{s_1 , s_2\}$ and $S_2 =\{s_1 , s_3\}$, we write

10 $$\begin{aligned} D=S_1\cup S_2 = \{s_1 s_2 | s_1 s_3\}. \end{aligned}$$

If a DO exhibits a distribution to only one subset of species, then this DO is an organization in the sense of COT. Otherwise, we call it a “*genuine DO*”.

Mathematically, the significance of DOs is proven by the fact that the set of persistent species of every solution of a reaction-diffusion system is always a DO^20^.

**Figure 1:**
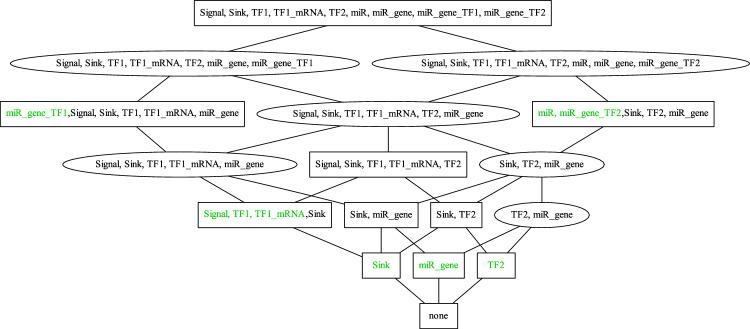
Lattice of DOs of micro-RNA transcription-factor interaction model from^23^. The 17 vertices of the lattice represent the DOs of the model. Each vertex displays the species of the respective DO. The boxes mark DOs that are organizations whereas the 6 ellipses mark genuine DOs. Species that do not appear in any DO that is a subset of that DO are marked green. The smallest DO of the lattice (which always has to be an organization) is at the bottom of the lattice and is empty since there is no inflow reaction in this example. Note, that in this figure there is no information contained about which reactions are active in the DOs. Note also, that each of the species $TF2, miR\_gene$ and *Sink* alone does not trigger any reaction. Therefore, these species create multiple DOs that are non-reactive (like the empty set). At the top of the lattice is the biggest DO. For this example, it contains all species of the model.

From a given reaction network the set of DOs can be computed without the need for any knowledge about the kinetics (reaction constants, kinetic laws applied, etc.). The set of DOs is always a lattice^20^. The lattice of DOs of the micro-RNA transcription-factor interaction model^23^ is shown in Fig. 1.

**Figure 2:**
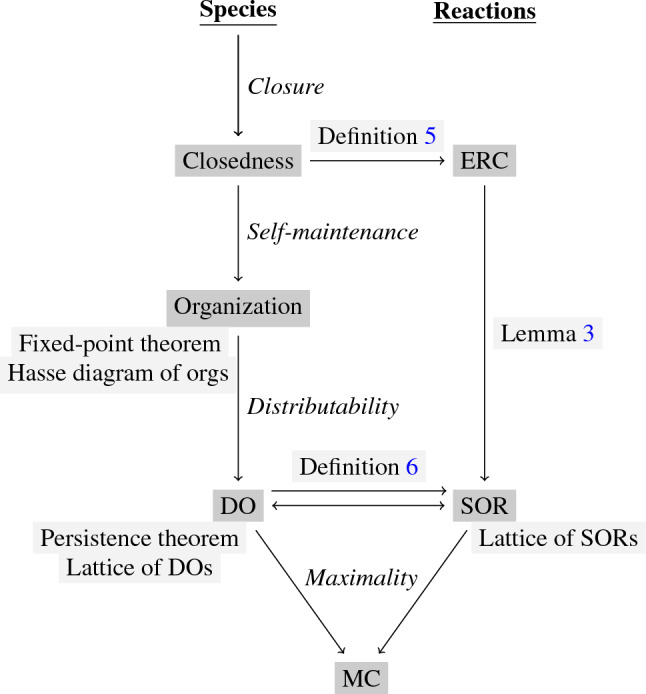
Overview of the main definitions of this work and their mutual interrelations. The boxes highlighted in gray are linked to the respective parts of the text where they are described. On the left-hand side, the items based on species are listed as presented in the Preliminaries of this work. On the right-hand side, the new reaction-based terms are listed as introduced in the Results of this work. The term MC stands for the maximal compartment and is defined in Definition 7.

The left-hand side of Fig. 2 shows the main definitions of this subsection together with their relations to one another.

Note, that these definitions are based upon subsets of species. In the next section, these definitions are complemented by the definitions and theorems (displayed on the right-hand side of Fig. 2) necessary for the algorithm to compute DOs. The ladder are based upon subsets of reactions.

## Results

### Novel theoretical concepts

In this section, the new definitions which are necessary to formulate the algorithm are stated.

#### Set of organizational reactions (SORs)

In principle, this is a transfer of the species-based definitions from above to a reaction-based approach. The first idea is to transfer Definition 1 of a closure of a subset of species to reactions.

##### Definition 5

(Elementary Reaction Closure (ERC)) Given a reaction network $({\mathscr {S}},{\mathscr {R}})$ and a reaction $\hat{r}\in {\mathscr {R}}$, we call the set

11 $$\begin{aligned} ERC(\hat{r})\equiv \{r\in {\mathscr {R}}:\,supp(r)\subseteqq clos(supp({\hat{r}}))\} \end{aligned}$$

of reactions the elementary reaction closure (ERC) of $\hat{r}$, that is, the set of reactions that are activated as soon as $\hat{r}$ is active.

By design, the ERC of a reaction is unique. In the implementations of the algorithm, the computation of the ERC of a reaction is realized by the function create_ERCs(). For the micro-RNA transcription-factor interaction model, Table 1 shows the ERC of each reaction.

**Table 1:** ERC of each reaction of the micro-RNA transcription-factor interaction model^23^.

Reaction	ERC
$r_1$	$r_1, r_2, r_{11}$
$r_2$	$r_2,r_1,r_{11}$
$r_3$	$r_3,r_4,r_5,r_6$
$r_4$	$r_4,r_3,r_5,r_6$
$r_5$	$r_5,r_4,r_6,r_2$
$r_6$	$r_6$
$r_7$	$r_7,r_8,r_{10},r_{11}$
$r_8$	$r_8,r_{10},r_{11}$
$r_{9}$	$r_9$,$r_6$,$r_8,r_{10},r_{11}$
$r_{10}$	$r_{10},r_8,r_{11}$
$r_{11}$	$r_{11}$

Here the elements of each ERC are listed in the order of their computation even though mathematically an ERC is a set.

In the following, the recursive construction of the ERC of $r_3$ is described as an example. The species $miR\_gene$ and *TF*2 are required to run reaction $r_3$, which produces species $miR\_gene\_TF2$. This in turn triggers the reactions $r_4$ and $r_5$. This results in the addition of the species $miR\_gene$ and *miR*. *miR* triggers the reaction $r_6$, thus the reaction $r_6$ is added at last. Since no further reactions are supported by the listed species set $\{miR\_gene, TF2, \}$ the ERC of $r_3$ is $r_3,r_4,r_5,r_6$.

Definition 6 transfers DOs, which were defined for species sets, to sets of active reactions.

##### Definition 6

Given a reaction network $({\mathscr {S}},{\mathscr {R}})$, a DO $D\subseteq {\mathscr {S}}$, and a feasible flux $\hat{v}\in {\mathbb {R}}_+^m$ with respect to *D*, such that *D* is a DO through ${\hat{v}}$, then:

- The set
  12 $$\begin{aligned} SOR({\hat{v}} )\equiv \{r_j\in {\mathscr {R}}:\,\hat{v}_j>0\} \end{aligned}$$
  is called *set of organizational reactions* (through $\hat{v}$) or shortly *SOR*.
- A subset $S\in \{S_1,\ldots ,S_k\}$ of species is called *compartment of the DO-SOR pair* $(D,SOR(\hat{v}))$ if there are distinct sets $S_1,\ldots ,S_k$ of *D* with
  13 $$\begin{aligned} \cup _{i=1}^k \{r\in {\mathscr {R}}:\, supp(r)\subseteqq S_i\}=SOR(\hat{v}). \end{aligned}$$
- A set $\{S_1,\ldots ,S_l\}$ of compartments of the DO-SOR pair $(D,SOR(\hat{v}))$ is called *compartmentalization of the DO-SOR pair* $(D,SOR(\hat{v}))$ if
  14 $$\begin{aligned} \cup _{i=1}^l S_i = D. \end{aligned}$$
  and
  15 $$\begin{aligned} \cup _{i=1}^l \{r\in {\mathscr {R}}:\, supp(r)\subseteqq S_i\}=SOR(\hat{v}). \end{aligned}$$
- A species $s_i\in {\mathscr {S}}$ is *overproduced* with respect to the flux vector $\hat{v}$ if
  16 $$\begin{aligned} (N{\hat{v}})_i>0. \end{aligned}$$

Definition 6 implies that for a given DO there is a unique maximal SOR that can be computed by the algorithm presented in this work.

##### Lemma 1

(Unique Set of Overproduction) To each $SOR({\hat{v}})$ belongs a unique biggest set of species that can be overproduced by the set of reactions contained in $SOR({\hat{v}})$.

##### Proof

There can be a number of flux vectors $\hat{v}_{1},\ldots , \hat{v}_{k} \in {\mathbb {R}}^m$, each tracing to $SOR(\hat{v})= \{r_j\in {\mathscr {R}}:\,\hat{v}_j>0\}$, but with different sets of overproduced species. Unifying all these sets of overproduced species results in a unique biggest set of overproduced species. $\square$

Roughly speaking, for a given DO, there can be multiple corresponding SORs, and conversely, for a given SOR, there can be multiple corresponding DOs. More precisely:

1. Given a reaction network $({\mathscr {S}},{\mathscr {R}})$ and a DO $D\subseteqq {\mathscr {S}}$, there can be several vectors $\hat{v}_{1},\ldots , \hat{v}_{k} \in {\mathbb {R}}^m_+$, through which a subset *D* is a DO. Nevertheless, the sets $SOR(\hat{v}_1),\ldots ,SOR(\hat{v}_k)$ of reactions can be different from each other. These differences describe the different potential behaviors of the DO *D* in terms of the active reactions. An example of a DO performing several SORs is indicated by a red link between SORs in the hasse diagram.
2. Given a reaction network $({\mathscr {S}},{\mathscr {R}})$, a set $D\subseteqq {\mathscr {S}}$ of species and a vector $\hat{v}\in {\mathbb {R}}^m_+$ such that *D* is a DO through $\hat{v}$. Then there can be different DOs all exhibiting the same behavior, that is, the same set $SOR(\hat{v})$ of active reactions. These DOs include one, say $D_{min}$, which is minimal in terms of its number of species and its number of compartments. The other DOs are unions of $D_{min}$ and further non-reacting species.

##### Lemma 2

(SORs form a lattice) The set of all SORs of a given reaction network $({\mathscr {S}},{\mathscr {R}})$ forms a lattice.

##### Proof

A lattice is a partially ordered set in which every two elements have a unique supremum (a least upper bound) and a unique infimum (a greatest lower bound).

1. *Partial order of the set of SORs*: The subset relation for sets provides a partial order.
2. *Unique supremum*: Given two SORs $R_1,R_2 \subseteq {\mathscr {R}}$, derived from two species subsets $D_1,D_2\subseteqq {\mathscr {S}}$ which are DOs through the vectors $\hat{v},\hat{\hat{v}}\in {\mathbb {R}}_+^m$, we consider
  17 $$\begin{aligned} R_{sup}\equiv R_1 \cup R_2 . \end{aligned}$$
  $R_{sup}$ is a SOR since $D\equiv D_1 \cup D_2$ is a DO through the vector $\hat{v}+\hat{\hat{v}}$. Thereby a compartmentalization of *D* is assumed, where the compartments of $D_1$ and $D_2$ are simply put next to each other disjointly without changing or merging them in any way. With that, the minimality of $R_{sup}$ follows trivially since no new reaction is activated and thus no further species can be attained.
3. *Unique infimum*: Given two SORs $R_1,R_2\subseteq {\mathscr {R}}$, we take the union of all SORs contained in $R_1 \cap R_2$ as infimum that is,
  18 $$\begin{aligned} R_{inf}\equiv \cup \{R\subseteq R_1 \cap R_2 :\; \text {R is a SOR}\}. \end{aligned}$$
  The union is finite and it is performed disjointly like the union of $R_1$ and $R_2$ to get a unique supremum. The existence of $R_{inf}$ follows from the fact that there exists a unique minimal SOR $R_{min}$ of the reaction network. This is the set of inflow reactions together with the reactions in the ERCs of the inflow reactions. The reactions of $R_{min}$ are included in any SOR. Note that $R_{min}$ can be empty. The uniqueness of $R_{inf}$ can be proven easily by contradiction: If there were two different greatest infima, unifying them would give a greater one in contradiction to the assumption.

$$\square $$

**Figure 3:**
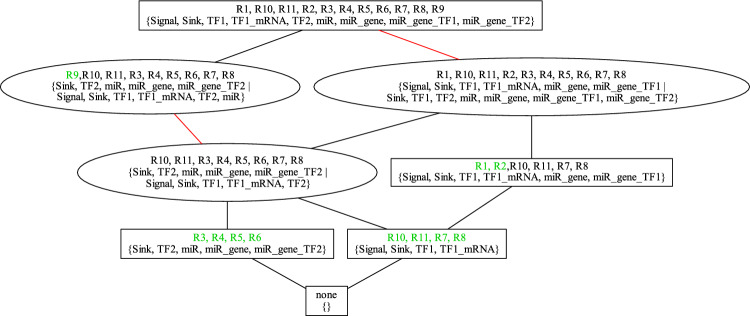
Lattice of the 8 SORs of the micro-RNA transcription-factor interaction model^23^. The boxes mark SORs that can occur in an organization whereas the ellipses mark SORs that can occur in genuine DOs. A red line links two SORs representing the same species set. A reaction of a SOR is marked green if it is not contained in any SOR which is a subset of that SOR. Below the reactions of each SOR, the minimal corresponding DO $D_{min}$ is given. A more detailed description of all information on the lattice is given in Section “Interpreting a lattice of SORs”.

The lattice of SORs of the micro-RNA transcription-factor interaction model^23^ is shown in Fig. 3.

For models of Influenza-A and SARS-CoV-2 virus infection dynamics, in^24^ resp.^25^ it was shown how such lattices can be used to better understand model dynamics and and also to compare different models. Lemma 3 provides an equivalent definition of SORs that use ERCs to guarantee closedness and does not explicitly refer to DOs. It is applied in the implementation of the algorithm to compute SORs.

##### Lemma 3

(Equivalent Definition of SORs) Given a reaction network $({\mathscr {S}},{\mathscr {R}})$, a subset $R\subseteqq {\mathscr {R}}$ of reactions is a SOR if and only if there is a vector $\hat{v}\in {\mathbb {R}}_+^m$ with the following two properties:

1. Self-maintenance: $N\hat{v}\ge 0$ and
2. Closedness: $$R=\cup \{ERC(r_j):\,\hat{v}_j>0\}$$.

##### Proof

Following Definition 6 it suffices to show that there as an appropriate corresponding DO $D=\cup _{i=1}^k S_i$ with closed subsets $S_1,\ldots ,S_k\subseteqq {\mathscr {S}}$ of species and feasible fluxes $\hat{v}^1,\ldots ,\hat{v}^k$ with respect to each $S_i$ such that $\hat{v}=\sum _{i=1}^k \hat{v}^i$.

Let $k\equiv |R|$, that is, $R=\{r_1,\ldots ,r_k\}$. For each $i=1,\ldots ,k$, subsets

19 $$\begin{aligned} S_i\equiv clos(supp(r_i)) \end{aligned}$$

of species are defined which are closed by definition. Thus, there is an equivalence between the set of species subsets $S_i$ and the set of elementary reaction closures $ERC(r_j)$.

For each $j=1,\ldots ,k$, the number

20 $$\begin{aligned} l(j)\equiv \{i\in \{1,\ldots ,k\}:\,supp(r_j)\subseteqq S_i\}, \end{aligned}$$

is defined which is the number of species subsets $S_i$ supporting the reaction $r_j$. Furthermore, for each subset $S_i,\,i=1,\ldots ,k,$ and each reaction $r_j\in R,\,j=1,\ldots ,m,$ the number.

21 $$\begin{aligned} l(i,j)= {\left\{ \begin{array}{ll} 0, \text { if }supp(r_j)\nsubseteqq S_i \\ 1, \text { if }supp(r_j)\subseteqq S_i, \end{array}\right. } \end{aligned}$$

is defined which equals one if $r_j$ is supported by $S_i$, and which equals 0 if $r_j$ is not supported by $S_i$. Now, for each subset $S_i,\,i=1,\ldots ,k,$ and each reaction $r_j\in {\mathscr {R}},\,j=1,\ldots ,m,$ the feasible fluxes are defined by

22 $$\begin{aligned} \hat{v}^i_j= {\left\{ \begin{array}{ll} \dfrac{l(i,j)}{l(j)}\hat{v}_j, \text { if } r_j\in R\\ 0, \text { otherwise}. \end{array}\right. } \end{aligned}$$

$$\square $$

#### Maximal compartments (MCs) and minimal compartmentalizations

For real-life systems, evolutionary aspects such as efficiency are important. Efficiency can be realized by minimizing the number of compartments such systems exhibit. Definition 7 provides a description of the maximal compartments which can be realized for a given DO-SOR pair.

##### Definition 7

(Maximal Compartment (MC) and Minimal Compartmentalization of a DO-SOR pair) Given a reaction network $({\mathscr {S}},{\mathscr {R}})$, a DO $D\subseteqq {\mathscr {S}}$ and one of its associated SORs $R\subseteqq {\mathscr {R}}$, then:

- A compartment $S\subseteqq {\mathscr {S}}$ of the DO-SOR pair (*D*, *R*) is called *maximal* if there is no other compartment $\hat{S}$ of the DO-SOR pair (*D*, *R*) such that $S\subset \hat{S}$.
- A compartmentalization $\{S_1,\ldots ,S_l\}$ of the DO-SOR pair (*D*, *R*) is called *minimal* if its number *l* of compartments is minimal, that is, there is no compartmentalization of the DO-SOR pair (*D*, *R*) with a number of compartments lower than *l*.

The algorithm presented in this work can compute all MCs of a DO-SOR pair. The algorithm exploits the fact, that an MC is closed and does not support a reaction outside of the SOR. From the set of MCs of a DO-SOR pair, the algorithm can also compute minimal compartmentalization by selecting a subset of the set of MCs of the DO-SOR pair.

In the micro-RNA transcription-factor interaction model^23^ the application of MCs on the SOR $\{TF1\_degradation, TF1\_mRNA\_degradation, TF1\_transcription,$ $TF1\_translation, miR\_degradation, miR\_gene\_TF2\_binding,$ $miR\_gene\_TF2\_release, miR\_synthesis\}$ can be observed. as the vertex $\{R10, R2, R3, R4, R5, R6, R7, R8, R9\}$ in the lattice of SORs, see Fig. 3. The algorithm produces 3 MCs:

- MC1: {Signal, Sink,TF2, TF1_mRNA,TF1}
- MC2: {Sink, miR_gene, TF2, miR_gene_TF2, miR}
- MC3: {Sink, TF2, miR, TF1}

The MCs 1 and 2 are able to perform the SOR resulting in a minimal number of compartments of 2. These compartments are not unique. One can remove the species *TF*2 from *MC*2 without impacting the active reactions. The reactions supported in an MC can also be impacted by removing a species of its support, but only if the support of these reactions is also in another active compartment.

The right-hand side of Fig. 2 shows the new definitions of this subsection and relates them to those from the Preliminaries, which can be found on the left-hand side.

#### Persistence theorem for SORs and further implications for the dynamics

Theorem 4 is a persistence theorem, which traces the persistence theorem for species (Theorem 3.25 in^20^) and finally transfers it to reactions.

##### Theorem 4

(The set of persistent reactions is a SOR) Given a RDS23and

- A connected domain $\Omega$ with a $C^2$ smooth boundary $\partial \Omega$ and $0<\int _\Omega dx<\infty$,
- An underlying reaction network containing $n=|{\mathscr {S}}|$ species and $m=|{\mathscr {R}}|$ reactions,
- Diffusion rates $d_i\ge 0,\,i=1,\ldots ,n,$ and
- A so-called flux vector function $v:\;{\mathbb {R}}^n_+ \rightarrow {\mathbb {R}}^m_+,\; c\mapsto v(c),$ that is
  1. Lipschitz continuous on every bounded subset of ${\mathbb {R}}_+^n$ and
  2. Feasible, that is, for every $c\in {\mathbb {R}}^n_+$ the vector *v*(*c*) is a feasible flux with respect to the set $\{s\in {\mathscr {S}}:\, c_s>0\}$ of species present in *c*.

Furthermore given a bounded solution

24 $$\begin{aligned} \hat{c}:\;\Omega \times {\mathbb {R}}_+\rightarrow {\mathbb {R}}^n,\;(x,t)\mapsto c(x,t). \end{aligned}$$

of the RDS 23 with:

- Derivatives $\dfrac{\partial \hat{c}}{\partial t}$ and $\dfrac{\partial ^2 \hat{c}}{\partial x^2}$ are each continuous with respect to *x* and *t*,
- $|\hat{c}(x,t)|<K$ for all $x\in \Omega ,\,t\ge 0$ for a number $K\in {\mathbb {R}}$, that is, $\hat{c}$ is bounded,

then the set of persistent species of the solution $\hat{c}$ is a DO and the occurring compartmentalization is described by a flux vector $\hat{v}\in {\mathbb {R}}_+^m$ which is derived by double-integration with respect to *x* and *t* as defined in Lemma 3.23 of^20^.

From the definition of SORs (Definition 6) follows, that the set

25 $$\begin{aligned} \{{\textbf {r}}\in {\mathscr {R}}:\,\hat{v}_r>0\}, \end{aligned}$$

taken as the set of persistent reactions of the solution ${\hat{c}}$, is a SOR.

Therefore, by definition, a reaction is persistent if and only if the set of species in its support is persistent.

As shown in^21^, Theorem 4 does not only hold true for RDS but can also be transferred to the special cases of ODE and patch-like systems. Note that the concept of persistence used here is a new one that was defined in^20^. Note also that it is possible to extend Theorem 4 to other boundary conditions, for example, by adding appropriate reactions to the reaction network as it was indicated in^26^.

Theorem 4 also implies that a subset of reactions of the reaction network, which is not a SOR, can not persist. In other words, such a SOR must define a transient state of any solution of any RDS with that underlying reaction network.

From^20^ another implication for the dynamics, not in the long term but right after leaving the initial states, can be derived. Lemma 3.21 in^20^ states that for each compartment, the closure of the initially present species appears and does not vanish within a finite time. Transferred to reactions this means that right after leaving the initial state, in each compartment, the whole ERC of the initially active reactions is activated. Thus the set of reactions that persist is always a subset of the ERC of the initially active reactions.

### The algorithm

The algorithm for computing all sets of organizational reactions (SORs) and all distributed organization (DOs), representing the persistent subspaces, consists of two major steps: First, all elementary reaction closures (ERCs) are computed, and then SORs and DOs are computed by mixed integer linear programming (MILP). For MILP, the dimension of the search spaces is an upper bound for the complexity.

**Figure 4:**
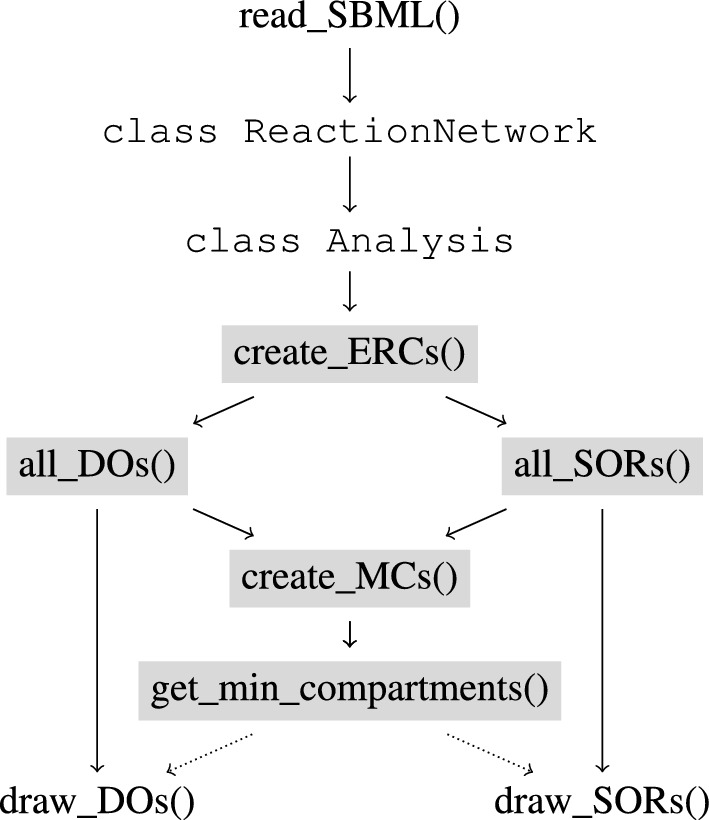
Main functions and classes of the algorithm and their relations to each other. To get directed to the descriptions of the functions click on their respective boxes.

In Fig. 4 the specific functions are shown interacting as a workflow including the most important data objects.

These functions are used jointly by the Analysis class. The functions create_MCs() and get_min_compartments() are used together in the function Analysis.get_compartmentalization_of_SOR_DO_pair(). Besides returning the output of the functions, the results are saved as attributes of the class as well. Further information about the usage of the classes and functions is available in the GitHub https://github.com/WoitkeL/dorganalysis.

#### Computing all elementary reaction closures (ERCs)

- Function: create_ERCs
- Input (required): a list of reactions
- Output: a dictionary mapping a reaction to its ERC

The algorithm originates straight-forward from the definition of closure of the reactions. For each given reaction, a species set for its ERC is created by unifying its support and its products. Any supported reactions and their products are added successively in a recursive function.

The result is saved as an instance of an ERC class object. The ERC class contains the attributes *reactions* and *species*. All the ERCs are then saved in an ERC-dictionary which is the means of the information transfer. Since in this work by assumption the total number *n* of species as well as the total number *m* of reactions are finite this also holds true for the highest order of all reactions

26 $$\begin{aligned} k\equiv max\{max\{|supp(r)|,|prod(r)|\}:\; r\in {\mathscr {R}}\}, \end{aligned}$$

which is lower or equal to the number of species *n*. Therefore, the function create_ERCs terminates and its time complexity is given in

27 $$\begin{aligned} {\mathscr {O}}(m^3 \cdot k ). \end{aligned}$$

Additionally, create_ERCs computes a transitive reduction^27^ to obtain a compact non-redundant representation of the ERCs. This reduces the number of constraints for the calculation of SORs and DOs. It is shown in that implementing the data as a matrix leads to a time complexity of the reduction of

28 $$\begin{aligned} {\mathscr {O}}(m^3). \end{aligned}$$

#### Computing all sets of organizational reactions (SORs)

- Function: all_SORs()
- Input: list of reactions
- Output: list of SORs

First, all ERCs are computed as described in the previous section. Then the largest SOR is computed by mixed-integer linear programming (MILP). All remaining SORs are obtained successively by integer cuts, excluding the SORs found so far.

For the MILP there are *m* continuous variables representing the flux vector $v\in {\mathbb {R}}_+^m$ and *m* discrete variables $b\in \{0,1\}^m$ denoting whether reaction $r_j$ is an element of the SOR ($b_j=1$) or not ($b_j = 0$). Thus, the search space is ${\mathbb {R}}_+^m \times \{0,1\}^m$. To find the largest SOR the following objective function is used

29 $$\begin{aligned} \max \sum _{j=1}^m b_j \end{aligned}$$

subject to the following constraints:

$$\begin{aligned}&N v\ge 0&\quad&\text {(self-maintenance)} \\&b_j \ge b_k \quad \forall r_k\in {\mathscr {R}}, \forall r_j\in ERC(r_k), r_k \ne r_j&\quad&\text {(reaction closure)} \\&b_j = 1 \hbox { for all inflow reactions}\ r_j \in {\mathscr {R}}&\quad&\text {(inflow)} \\&b_{j}\cdot c \ge v_{j}\ge b_{j} \quad \forall r_j\in {\mathscr {R}}&\quad&\text {(coupling variables} b_j) \end{aligned}$$

with the stoichiometric matrix $N\in {\mathbb {R}}^{n \times m}$ and the constant $c=10000$. Note that the variables are coupled such that $b_j = 1$ (reaction $r_j$ is part of the SOR) is equivalent to a strictly positive flux $v_j > 0$ of reaction $r_j$. An inflow reaction is a reaction with empty support and thus must be present in any SOR.

Solving the MILP has exponential time complexity since it is NP-hard^28^. The complexity for a single solution is exponential with the number of boolean variables, that is, the time complexity is given in

30 $$\begin{aligned} {\mathscr {O}}(2^m). \end{aligned}$$

When solving for several solutions there is a huge save in complexity when using Gurobi because of the use of a solution pool.

From the examples stated in Section “Interpreting a lattice of SORs” it will be clear, that the functions all_SORs() and all_DOs() exhibit the largest differences between the worst case runtime analyzed here and the actual runtimes. The latter is strongly impacted not only by the number of boolean variables but also by the number of constraints of the actual reaction network.

#### Computing all distributed organizations (DOs)

- Function: all_DOs()
- Input: list of reactions
- Output: list of DOs

Like for SOR computation, all ERCs are computed. Then the largest DO is computed by mixed-integer linear programming (MILP). All remaining DOs are obtained successively by integer cuts.

For the MILP there are *m* continuous variables representing the flux vector $v\in {\mathbb {R}}_+^m$ and *m* discrete variables $b\in \{0,1\}^m$ denoting whether reaction $r_j$ is active in the DO ($b_j=1$) and *n* discrete variables $e_j$ denoting whether species $s_i$ is an element of the DO ($e_i = 1$). Thus, the search space is ${\mathbb {R}}_+^m \times \{0,1\}^m \times \{0,1 \}^n$. To find the largest DO the following objective function is used

31 $$\begin{aligned} \max \sum _{i=1}^n e_i \end{aligned}$$

subject to the following constraints:

$$\begin{aligned}&N v\ge 0&\quad&\text {(self-maintenance)} \\&b_j \ge b_k \quad \forall r_k\in {\mathscr {R}}, \forall r_j\in ERC(r_k), r_k \ne r_j&\quad&\text {(reaction closure)} \\&b_j = 1 \text { for all inflow reactions} r_j \in {\mathscr {R}}&\quad&\text {(inflow)} \\&b_{j}\cdot c \ge v_{j}\ge b_{j} \quad \forall r_j\in {\mathscr {R}}&\quad&\text {(coupling variables} b_j)\\&b_j \ge e_i \quad \forall e_i\in {\mathscr {S}}, \forall r_j\in {\mathscr {R}}, supp(r_j) \subseteq clos(\{s_i\})&\quad&\text {(species closure)} \\&e_i \ge b_j \quad \forall e_i\in {\mathscr {S}}, \forall r_j\in {\mathscr {R}}, e_i \in supp(r_j) \cup prod(r_j)&\quad&\text {(coupling variables} e_i) \end{aligned}$$

Note that the variables are coupled such that if a reaction is present ($b_j = 1$) also all species involved as reactants or products must be present ($e_i = 1$). Further, note that the constraints ”species closure” ensure that a reaction must be present ($b_j = 1$) if it is supported in the single species closure of a species that is present ($e_i=1$).

Finally note that in the current implementation, Gurobi’s solution pool is used, which generates all DO-SOR pairs. Recall that one DO can have many SORs. From these solutions, only the DOs are returned. Internally, however, the tool stores all SORs in a DO-SOR dictionary for later use.

The worst case time complexity of all_DOs() is given in

32 $$\begin{aligned} {\mathscr {O}}(2^{m+n}), \end{aligned}$$

since compared to all_SORs() there is a further boolean variable for each of the *n* species.

#### Computing all maximal compartments (MCs)

- Function: create_MCs()
- Input: Reaction network, SOR, DO
- Output: list of MCs (species sets)

The function create_MCs() computes the (unique) maximal compartments of a given SOR. It works on a list of candidates for the MCs, which is initialized by the set of species of the DO and then broken down through the following four steps.

First, for each compartment it is checked if it supports an inactive reaction, that is, a reaction not contained in the SOR. If this is the case, the compartment is split into *k* compartments, where *k* is the order of the reaction. Each compartment is missing exactly one species of the support for the reaction. The statements regarding the time complexity are with the condition of disjoint supports of all reactions resulting in $k \cdot m \le n$ The time complexity of this first step is given in ${\mathscr {O}}(n\cdot k^{m+1}).$

After that, compartment candidates, which are subsets of other valid candidates, are eliminated: ${\mathscr {O}}(2^n\cdot 2^n)={\mathscr {O}}(4^n).$

Next, the candidates are checked for closedness. Each reaction, which extends the present species set can not occur in this set and is therefore inactivated by updating the MCs in the same way as done for the inactive reactions, that is, by splitting it. This check of closure is repeated until none of the compartments is altered. The total time complexity of the check for closure is given in ${\mathscr {O}}(m^2 \cdot 2^n \cdot k \cdot n).$

Finally, proper subsets of candidates are deleted, if present: ${\mathscr {O}}(4^n).$ Thus the total time complexity of create_MCs() is given in

33 $$\begin{aligned} {\mathscr {O}}(4^n). \end{aligned}$$

#### Computing a minimal compartmentalization of a SOR

- Function: get_min_compartments()
- Input: Reaction network, SOR, MC_list
- Output: list of MCs

The maximal compartments (MCs) listed in MC_list are used in an ILP to find the minimal number of compartments needed for the affiliated SOR. For each $MC\in MC\_list$ there is a discrete variable $b_{MC}\in \{0,1\}$ denoting whether the *MC* is part of the minimal compartmentalization ($b_{MC}=1$) or not ($b_{MC}=0$). The ILP, which is a set cover problem^29^, uses the objective function:

34 $$\begin{aligned} \min {\mathop \sum \limits _{MC \in MC\_list}}b_{MC} \end{aligned}$$

subject to the following constraints:

35 $$\begin{aligned} {\mathop \sum \limits _{MC\in MC\_list:\,species\in MC}}b_{MC}&\ge 1\text { for all species in species(SOR)}\end{aligned}$$

36 $$\begin{aligned} {\mathop \sum \limits _{MC\in MC\_list:\,supp(reaction) \subseteqq MC }} b_{MC}&\ge 1\text { for all reactions of the SOR} \end{aligned}$$

The first constraint ensures that all species are covered by the MCs and the second constraint guarantees that each reaction of the given SOR is active in at least one MC. With this, a minimal set of MCs, a minimal compartmentalization, is found such that all species in *species*(*SOR*) are covered and each reaction of the SOR can run in at least one MC. Since the problem resembles the set cover problem, which is proven NP-complete, it is not possible, to solve this problem in polynomial time and the LP seems to be the most efficient way to solve this.

Solving the ILP is of exponential time complexity, that is, given in

37 $$\begin{aligned} 2^{\text {number of MCs}}. \end{aligned}$$

### Analysis of the models of the bio models database

This section provides analyses of the models of the BioModels Database^30^ performed by using the algorithms presented in the previous chapter. At the end of this section, the lattice of SORs of an artificial reaction network, which is not contained in the BioModels database, is interpreted to exemplify which information can be drawn from it.

#### SORs, organizations and genuine DOs

932 models of the BioModels Database were transformed into a proper reaction network using the SBML reader of the libsbml package. These are analyzed in this section. To keep the study tractable, only the reactants and products of a reaction are considered, more precisely, the information contained in other elements of an SBML-model is ignored, for example, modifiers, rules, events, kinetic laws, or whether a species is flagged as constant.

**Figure 5:**
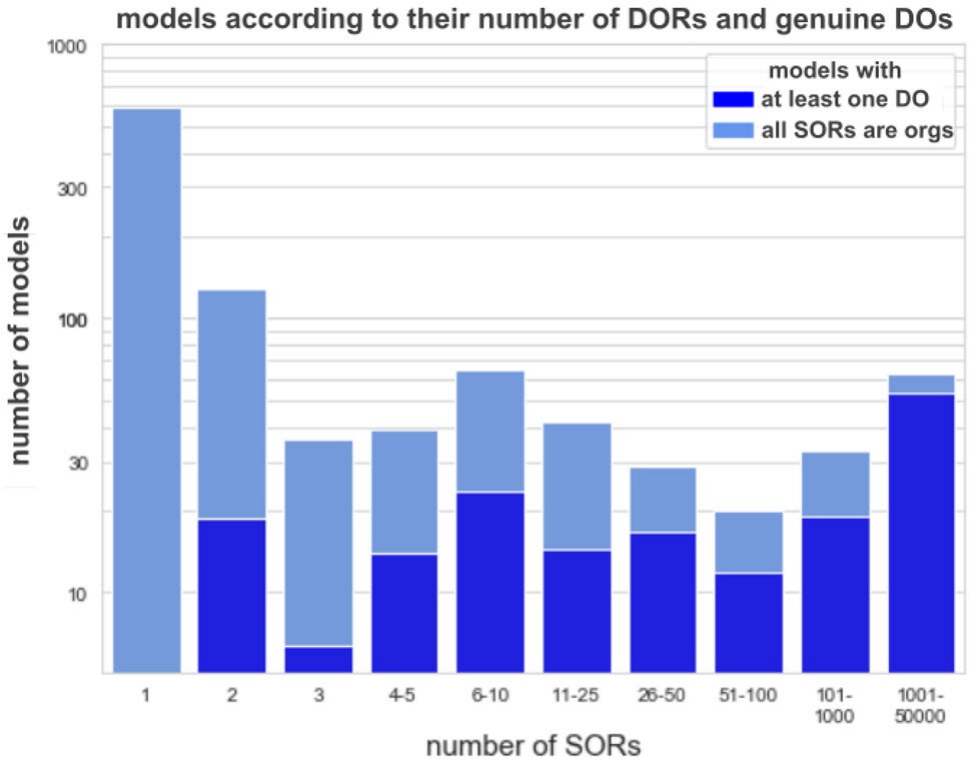
Log-scaled numbers of models according to their number of SORs and DOs resp. organizations. Models that have at least one SOR that is a genuine DO (i.e., that can not be represented by an organization) are marked dark blue and the others are marked light blue. The fraction equals zero for models with only one SOR confirming the theorem stating that every model has at least one organization^20^.

The github contains the file *biomodels.csv* with all extracted information of each model. In total, the algorithm computed 1’019’600 SORs for the 932 models, of which 218’870 can be represented by an organization, that is about $21.5\%$. All other SORs represent what are called genuine DOs, which are SORs that cannot be represented by organizations. In Fig. 5, all models are shown according to the number of their SORs and the fraction of SORs representing organizations.

Even though the majority of models do not exhibit any genuine DO, about four-fifths of all SORs are genuine DOs. More precisely, the fractions of models containing at least one genuine DO appear to increase with increasing number of SORs: $0\%$ (for models with only one SOR), $14\%$ (for models with two SORs), $17$, $36$, $36$, $34$, $57$, $59$, $57$, $79\%$ (for models with 1001 to 50,000 SORs). The reason for this is that with an increasing number of SORs also the number of possibilities for creating new genuine DOs (by combining SORs separately with each other) increases.

#### Separability of supports and order of reaction

**Figure 6:**
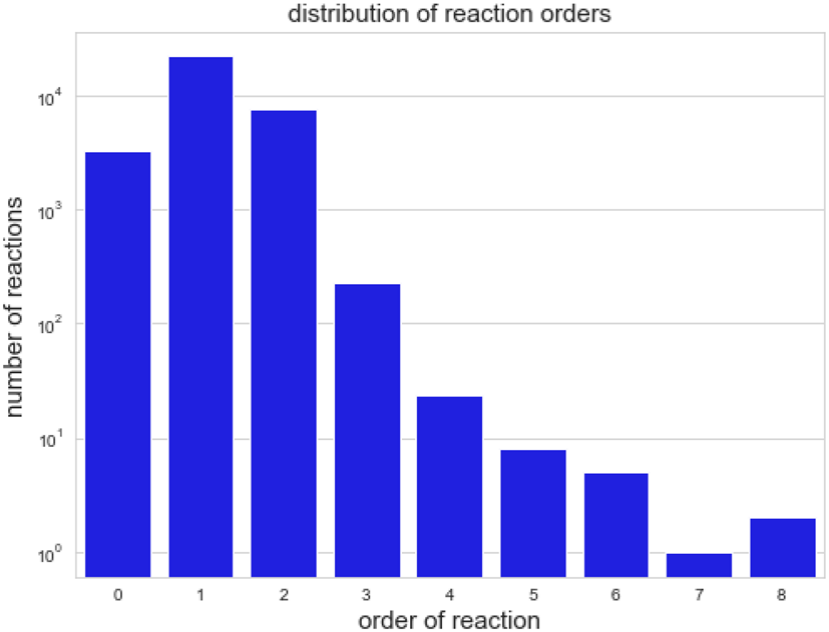
Log-scaled distribution of all reactions of the analyzed models of the BioModels Database with respect to their order. Reactions of order 1 dominate the set of models. Only $22.18\%$ of the reactions have an order bigger than one and therefore allow for separating the species into several compartments and attaining a genuine DO.

**Figure 7:**
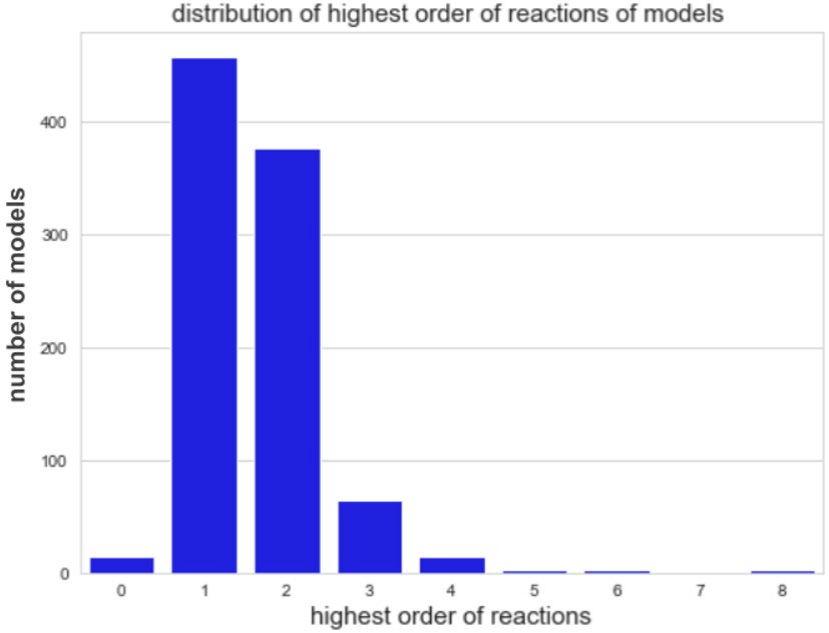
Distribution of the highest order of reactions for all analyzed models of the BioModels Database. There are only a few models containing only zero-order reactions. Models of reaction order at most 1 are found to dominate. $51.20\%$ of the models do not have any reaction of orders bigger than one and therefore are not capable of exhibiting a genuine DO.

Now the prerequisites for genuine DOs are studied on the level of reactions. This will make clear why most of the models cannot exhibit any genuine DO. Separability of the support of a reaction is a necessary condition for genuine DOs since it is a prerequisite for disabling a reaction. In turn, separability of the support of a reaction is possible only if the order of a reaction is greater than one, that is, the number of species of its support is at least two. The higher the order of a reaction the more ways there are to construct a genuine DO by distributing species and this in turn increases the probability of the appearance of a genuine DOs. Figures 6 and 7 provide an overview of the orders of reactions of the analyzed models of the BioModels Database.

Log-scaled distribution of all reactions of the analyzed models of

Only $14\%$ of all reactions have an order bigger than 1 and thus can be deactivated by separating their supports. Most of the models do not contain reactions of higher order. Thus most of the models cannot exhibit a genuine DO. This, together with the above-mentioned observation of genuine DOs often resulting from combining others, explains why only 237 of the 932 analyzed models contain at least one SOR that is a genuine DO. In the following the structure of the computed SORs is studied, i.e., their compartmentalizations or, more precisely, their numbers of compartments.

#### Minimal compartmentalizations

**Figure 8:**
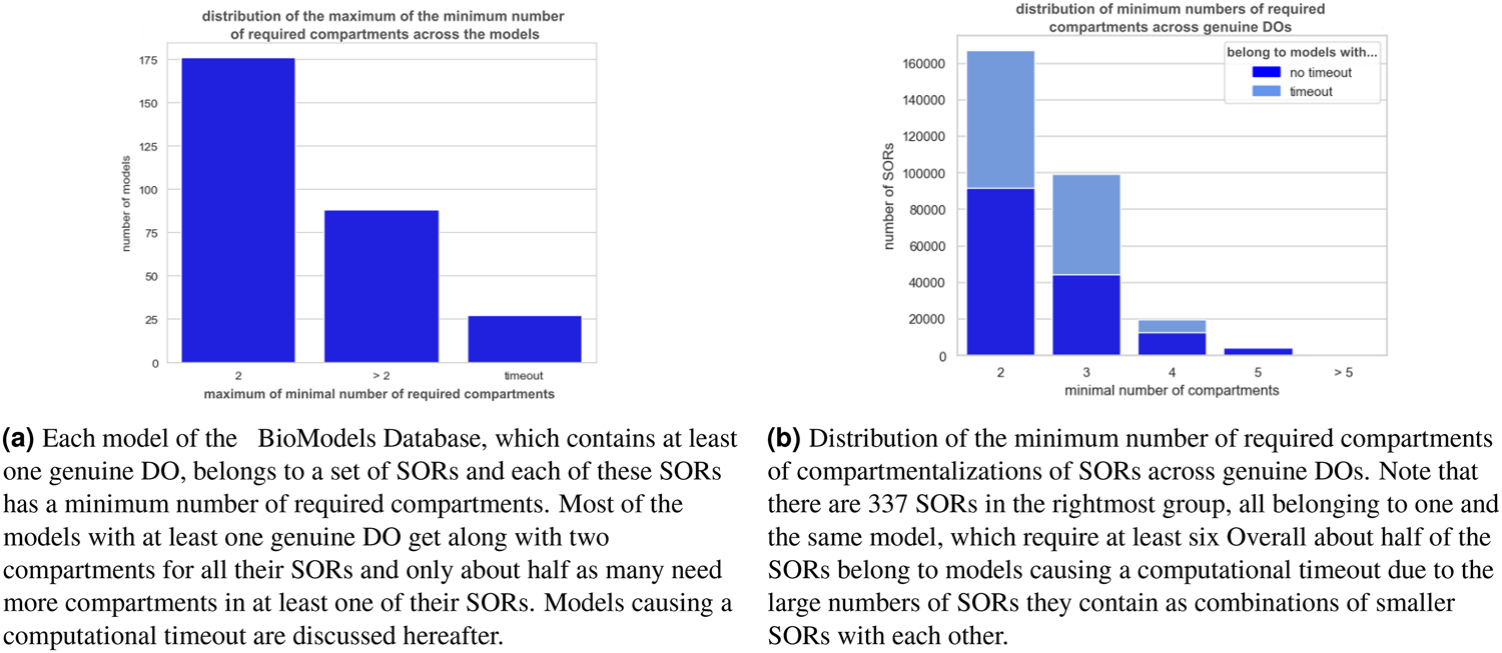
For all SORs requiring at least two compartments, that is genuine DOs: Distribution of the minimum number of required compartments across models (**a**) and across SORs (**b**). The 27 models causing timeout contain about half of all SORs. In fact, each model that causes a timeout contains well over 30,000 SORs.

For SORs requiring at least two compartments, that is, for genuine DOs, Fig. 8 provides an overview of the distribution of the minimum numbers of required compartments across the models (subfigure (a)) resp. the SORs (subfigure (b)).

Even though most models get along with one compartment for all their SORs there is a non-negligible number of models each containing at least one genuine DO (Fig. 8a). More precisely, SORs were found with their minimum number of required compartments occupying all numbers in the range from one to five (Fig. 8b). Here, the number of SORs decreases monotonously with an increasing minimum number of required compartments. Probably the reason for this is that the more compartments are needed, the more reactions have to be disabled simultaneously which in turn is even harder the more reactions have to be disabled.

#### Runtime and timeout analyses

**Figure 9:**
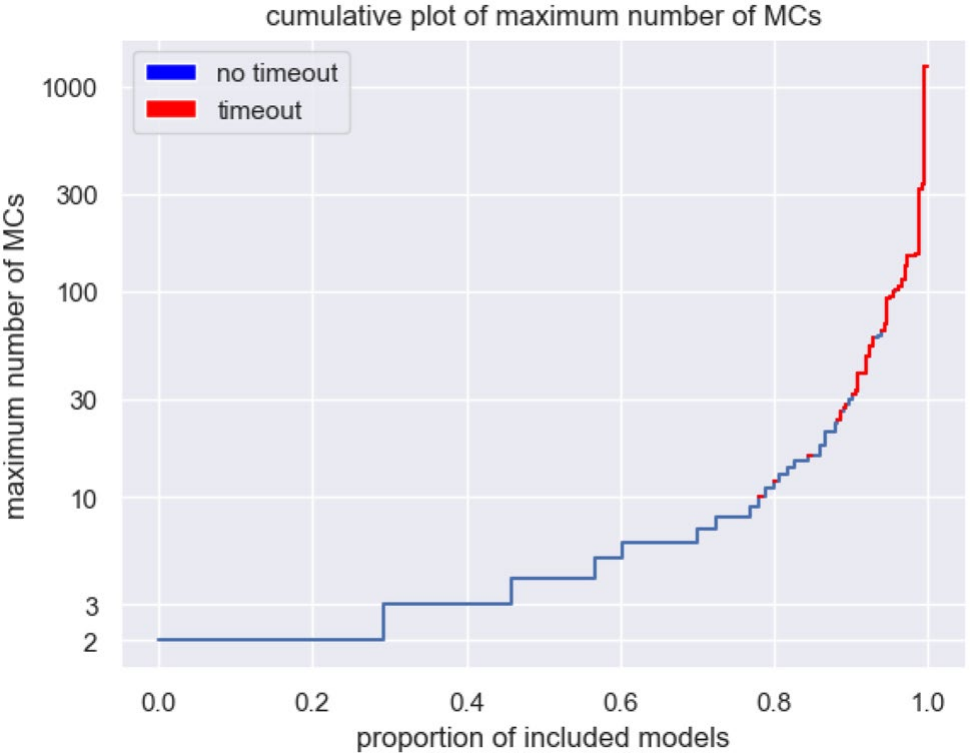
Each model of the BioModels Database, that contains at least one genuine DO, belongs to a set of SORs. Each of these SORs has minimal compartmentalization, which is a minimal set cover of the SOR consisting of a selection of maximal compartments (MCs) of this SOR. This diagram plots for each model the maximum number of MCs whose size is a very likely bottleneck of the algorithm causing a computational timeout (red lines).

**Figure 10:**
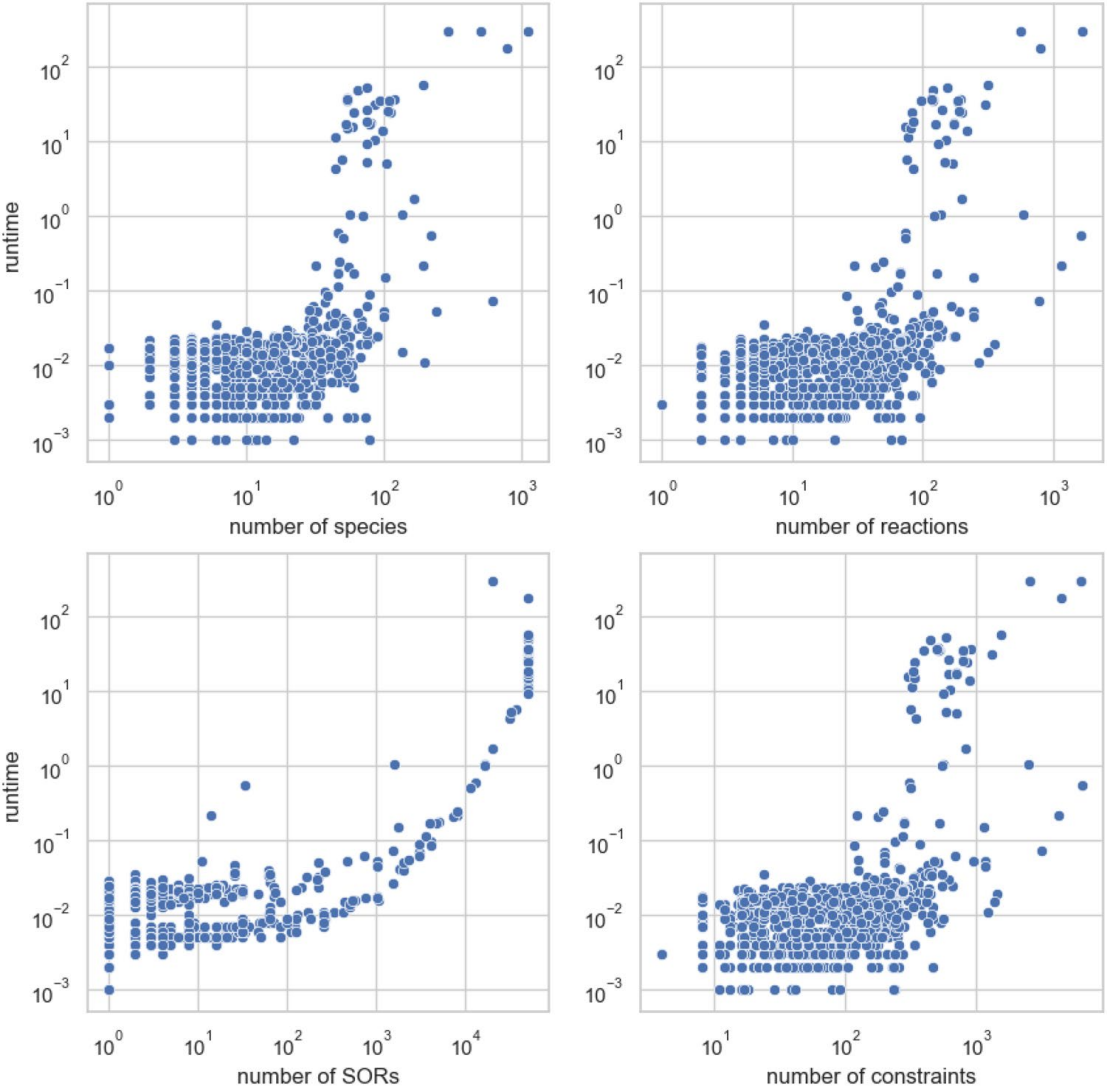
Runtime for each model depends on the following parameters: number of species, number of reactions, number of SORs and number of constraints applied in the LP to compute the SORs. Of these parameters, the number of SORs in a model appears to be the strongest indicator of runtime.

While analysing the BioModels Database, all 3 parts of the algorithm resulted in large running times for some models respectively. The calculation of ERC only came close to the timeout cap in two exceptionally large models with more than 1500 reactions (BIOMD0000000255,BIOMD0000000595). The calculation of SORs and DOs was a problem for 2 models with more than 500 reactions and reactions of a high order (BIOMD0000000255 and BIOMD0000000496). Figure 10 provides an overview of how the runtime of SOR computation for each model relates to the number of species, the number of reactions, the number of SORs, and the overall number of constraints of the LP used to compute the SORs. The upper limit of the number of SORs is given as parameter of the LP. The default is 50.000. Heuristic algorithms using a combinatorial build up, could be used to grasp all these basic SORs. A similiar approach is used for an alternative computation of the DOs from the set of SORs. This function is available in the *iterate_over_DB* file. Most of the models not fully processed arose from the computation of all minimal numbers of compartments. Figure 9 reveals the distribution of the maximum number of MCs across the models and its correlation to computational timeouts.

As the maximum number of MCs increases, the number of models becomes smaller and the probability of a computation timeout increases, making the maximum number of MCs a good indicator of runtime. Heuristic approaches or greedy algorithms governing the set cover problem could be implemented in that case (Fig. 10).

#### Interpreting a lattice of SORs

To conclude this chapter, an artificial 3-generator model is introduced to exemplify the output of the algorithm presented in this paper and to demonstrate how to interpret it. When limited to organizations, such outputs can be organized in Hasse diagrams, which were used previously to compare and hierarchize Influenza-A and SARS-CoV-2 infection dynamics models^24,25^. By generalizing organizations to DOs and SORs in this study, it was shown that the output of the algorithm is based on lattices of DOs resp. SORs.

The 3 generators, A1, A2 and A3, produce their corresponding products B1, B2 and B3 through the reactions R1, R2 and R3, respectively. The generator A2 is harmed by exposure to the products of the other generators, that is, B3 (reaction R4) and B1 (reaction R5). Thereby, also B1 is destroyed (R5). The harming of the production of B2 is counteracted by the reaction R6, which transforms B1 and B3 to two B2. Then there is an asymmetric reaction R7 erasing A1 and A2 when they meet. R7 is compensated by the last two reactions, namely R8 together with R9, which (through the production of the intermediate species D1) destroy the generators A1 and A3 and multiply B1 and thus also compensate for the destruction of B1 through R5. All nine reactions of the artificial model are listed below.

$$\begin{aligned} R1&: A1 \longrightarrow A1 + B1&\quad (\text {generating function 1})\\ R2&: A2 \longrightarrow A2 + B2&\quad (\text {generating function 2})\\ R3&: A3 \longrightarrow A3 + B3&\quad (\text {generating function 3})\\ R4&: A2 + B3 \longrightarrow B3&\quad (\text {destruction of A2})\\ R5&: B1 + A2 \longrightarrow \emptyset&\quad (\text {destruction of A2 and B1})\\ R6&: B1 + B3 \longrightarrow 2B2&\quad (\text {product interaction benefiting B2})\\ R7&: A1 + A2 \longrightarrow \emptyset&\quad (\text {generator interaction weakening A1 and A2})\\ R8&: A1 + A2 + A3 \longrightarrow D1&\quad (\text {production of D1})\\ R9&: D1 + B1 \longrightarrow 2B1 + A2&\quad (\text {production of B1, A2 compensates R5, R7}) \end{aligned}$$

**Figure 11:**
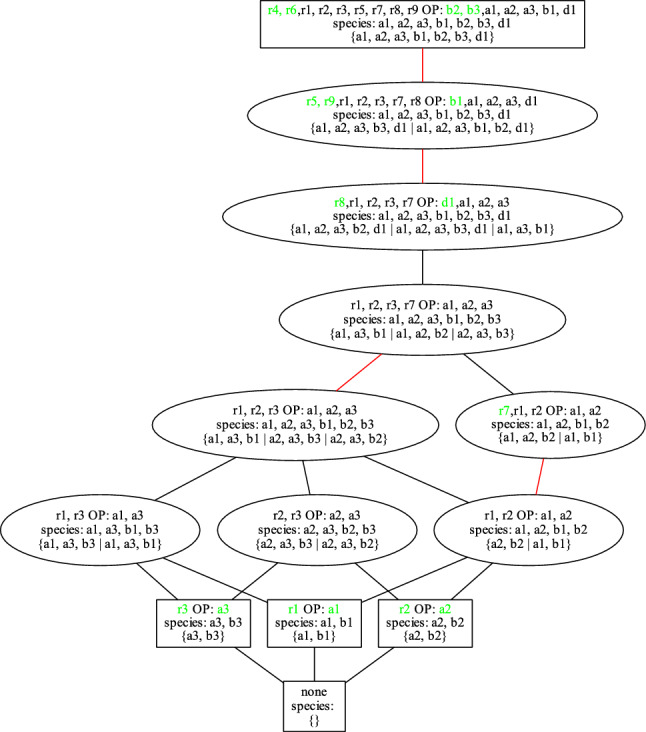
The lattice of the 13 SORs of the artificial 3-generator model. For each SOR *S*, the corresponding DO *D* with the minimal compartmentalization is considered to provide the following information about *S* in its node: in the first line: reactions of *S* and overproduced (OP) species, in the second line: species of *D* and in the third line: minimal compartmentalization. A reaction resp. species of *S* is marked green if it is new, that is, if it does not appear in any SOR that is a subset of *S*. If there is only one compartment, then *D* is an organization and the border of the node is a rectangle (instead of an ellipse). Two SORs are linked by a line if their reactions form subsets and there is no other SOR between them. Such a line is red to indicate that the two linked SORs have the same set of species in their respective DOs. This marks different activation/deactivation patterns of reactions solely caused by different compartmentalizations of one and the same set of species.

The lattice of all SORs of this artificial 3-generator model computed with compute_all_SORs() together with further information about the minimal compartmentalizations is shown in Fig. 11.

Only the largest SOR as well as the four smallest SORs of the artificial 3-generator model represent organizations whereas the intermediate SORs represent genuine DOs, which display different levels of compartmentalization of one and the same set of species. All three generators can exist independently of each other and build up the first level of reactive compartments (2nd line of the lattice when viewed from the bottom). They can be combined to build the first level of SORs that can only be obtained as genuine DOs as can be seen from the round shape of the nodes.

The outflow reaction R7 can then be activated by putting the generators A1 and A2 in one and the same compartment. Optional reactions like these can inflate the number of SORs, but here this is not the case since the support of R7 is a subset of the supports of the reactions that are required for larger SORs, which in turn keeps the lattice slim and easier to interpret.

The three SORs directly below the largest SOR require at least three compartments. This is quite a rare occurrence when compared with the results of the BioModels database (see Fig. 8a). This is because there has to be a specific pattern of reactions so that neither compartment can be merged with another one. To make these 3 possible merges impossible, a minimum number of higher-order reactions is required.

Note, that the artificial 3-generator model is the default network of the algorithm if no SBML file is handed over to it.

## Conclusion

In this work, an algorithm was presented for the first time that allows computing all persistent subspaces of a reaction-diffusion system (RDS) from the underlying reaction network alone, i.e., without knowledge of kinetic details. For this purpose, the theory developed in^20^ has been extended from the species (or variable) level of the RDS to the reaction level by appropriate mathematical definitions. The main connections between these new definitions were presented and proved, culminating in the persistence Theorem 4 for SORs, which characterizes all persistent subspaces of an RDS at the level of reactions and allows for describing their inner structure in a mathematically precise way.

Then, the algorithm was presented which allows computing all SORs and DOs of an RDS by linear programming, as well as describing their possible internal structure in terms of compartmentalizations. An upper bound for the runtime was given for each part of the algorithm. An implementation of the algorithm in Python including documentation for the application is freely available in the Github https://github.com/WoitkeL/dorganalysis.

Finally, the algorithm was applied to all models of the BioModels Database to analyze them with respect to the occurrence of genuine DOs and their structure and to practically test the performance of the algorithm. This confirmed both the importance of DOs in general and that of genuine DOs for the analysis of RDS, as well as demonstrating the practicality of the algorithm and its implementation.

Thus, a framework is now available that allows RDS to be studied both mathematically-theoretically through a kind of generalized fixed point analysis and practically by computing all DOs or SORs to analyze, evaluate, construct, and compare RDS models from different disciplines such as chemistry, biochemistry, ecology, or sociology.

Future research tasks following this work include e.g. a further extension and generalization of the mathematical theory developed here e.g. for unconstrained systems, the application of the algorithm developed here to concrete models from different disciplines including a more detailed analysis and interpretation of the models of the BioModels Database especially with respect to the function of genuine DOs and their biological meaning, and a more detailed analysis of the runtime and complexity of the presented algorithm including possible improvements.

## Data Availability

An implementation of the algorithm developed in this work is available in the GitHub https://github.com/WoitkeL/dorganalysis.

## References

[1] Aris R (1965). Prolegomena to the rational analysis of systems of chemical reactions. Arch. Ration. Mech. Anal..

[2] Bailey JE (2001). Complex biology with no parameters. Nat. Biotechnol..

[3] Kreyssig P (2014). Effects of small particle numbers on long-term behaviour in discrete biochemical systems. Bioinformatics.

[4] Ibrahim B (2013). Spatial rule-based modeling: A method and its application to the human mitotic kinetochore. Cells.

[5] Ibrahim B (2015). Toward a systems-level view of mitotic checkpoints. Prog. Biophys. Mol. Biol..

[6] Ibrahim B, Dittrich P, Diekmann S, Schmitt E (2007). Stochastic effects in a compartmental model for mitotic checkpoint regulation. J. Integr. Bioinform..

[7] Jiang C, Szymanski BK, Lian J, Havlin S, Gao J (2021). Nuclear reaction network unveils novel reaction patterns based on stellar energies. New J. Phys..

[8] Hufsky F (2021). Computational strategies to combat COVID-19: Useful tools to accelerate SARS-CoV-2 and coronavirus research. Brief. Bioinform..

[9] Matsumaru N, Centler F, di Fenizio PS, Dittrich P (2006). Chemical organization theory applied to virus dynamics. it Inf. Technol..

[10] Henze R, Dittrich P, Ibrahim B (2017). A dynamical model for activating and silencing the mitotic checkpoint. Sci. Rep..

[11] Ibrahim B (2018). A new era of virus bioinformatics. Virus Res..

[12] Petri, C. A. Kommunikationen mit Automaten. Ph.D. thesis, PhD Thesis, University of Bonn (1962).

[13] Veloz T (2020). The complexity-stability debate, chemical organization theory, and the identification of non-classical structures in ecology. Found. Sci..

[14] Dittrich, P. & Winter, L. Reaction networks as a formal mechanism to explain social phenomena. In *Proceeding of The Fourth International Workshop on Agent-based Approaches in Economics and Social Complex Systems (AESCS 2005)* (eds. Deguchi, H., Kijima, K., Terano, T., Kita, H.) 433–446 (2005).

[15] Dittrich P, Winter L (2008). Chemical organizations in a toy model of the political system. Adv. Complex. Syst..

[16] Dittrich P, Di Fenizio PS (2007). Chemical organisation theory. Bull. Math. Biol..

[17] Henze R (2019). Multi-scale stochastic organization-oriented coarse-graining exemplified on the human mitotic checkpoint. Sci. Rep..

[18] Peter S, Dittrich P (2011). On the relation between organizations and limit sets in chemical reaction systems. Adv. Complex. Syst..

[19] Tyson J (1991). Modeling the cell division cycle: cdc2 and cyclin interactions. Proc. Natl. Acad. Sci. U. S. A..

[20] Peter S, Ibrahim B, Dittrich P (2021). Linking network structure and dynamics to describe the set of persistent species in reaction diffusion systems. SIAM J. Appl. Dyn. Syst..

[21] Ibrahim B, Peter S (2023). Persistent subspaces of reaction-based dynamical systems. MATCH Commun. Math. Comput. Chem..

[22] Granas A, Dugundji J (2003). Fixed Point Theory.

[23] Proctor CJ, Smith GR (2017). Computer simulation models as a tool to investigate the role of micrornas in osteoarthritis. PLoS ONE.

[24] Peter S (2019). Structure and hierarchy of influenza virus models revealed by reaction network analysis. Viruses.

[25] Peter S, Dittrich P, Ibrahim B (2021). Structure and hierarchy of SARS-CoV-2 infection dynamics models revealed by reaction network analysis. Viruses.

[26] Peter S, Ghanim F, Dittrich P, Ibrahim B (2020). Organizations in reaction-diffusion systems: Effects of diffusion and boundary conditions. Ecol. Complex..

[27] Hsu HT (1975). An algorithm for finding a minimal equivalent graph of a digraph. J. ACM.

[28] Papadimitriou CH, Steiglitz K (1998). Combinatorial Optimization: Algorithms and Complexity.

[29] Vazirani VV (2001). Approximation Algorithms.

[30] Malik-Sheriff RS (2020). BioModels—15 years of sharing computational models in life science. Nucleic Acids Res..

